# Author Correction: Human SARS-CoV-2 has evolved to reduce CG dinucleotide in its open reading frames

**DOI:** 10.1038/s41598-021-91385-y

**Published:** 2021-06-03

**Authors:** Yong Wang, Jun‑Ming Mao, Guang‑Dong Wang, Zhi‑Peng Luo, Liu Yang, Qin Yao, Ke‑Ping Chen

**Affiliations:** 1 School of Food and Biological Engineering, Jiangsu University, 301 Xuefu Road, Zhenjiang, 212013 China; 2 Institute of Life Sciences, Jiangsu University, 301 Xuefu Road, Zhenjiang, 212013 China

Correction to: *Scientific Reports* https://doi.org/10.1038/s41598-020-69342-y, published online 23 July 2020

The original version of this Article incorrectly stated that coronaviruses have the largest of all reported RNA genomes. This statement has now been corrected for accuracy. In the Introduction,

"Coronaviruses have the largest RNA genomes among all viruses."

now reads

"Coronaviruses have some of the largest RNA genomes among all viruses."

The original Article has been corrected.

